# Analysis of national surveillance of respiratory pathogens for community-acquired pneumonia in children and adolescents

**DOI:** 10.1186/s12879-022-07263-z

**Published:** 2022-04-04

**Authors:** Eui Jeong Roh, Mi-Hee Lee, Ji Young Lee, Hyo-Bin Kim, Young Min Ahn, Ja Kyoung Kim, Hyoung Young Kim, Sung-Su Jung, Minji Kim, Eun Kyeong Kang, Eun-Ae Yang, Soo Jin Lee, Yang Park, Ju-Hee Seo, Eun Lee, Eun Seok Yang, Kang Seo Park, Meeyong Shin, Hai Lee Chung, Yoon Young Jang, Bong Seok Choi, Jin-A. Jung, Seung Taek Yu, Myongsoon Sung, Jin Tack Kim, Bong-Seong Kim, Yoon Ha Hwang, In-Suk Sol, Hyeon-Jong Yang, Man Yong Han, Hae Young Yew, Hyoung Min Cho, Hye-young Kim, Yeon-Hwa Ahn, Eun Sil Lee, Dong Hyeok Kim, Kyujam Hwang, Sang Oun Jung, Jung Yeon Shim, Eun Hee Chung

**Affiliations:** 1 Department of Pediatrics, Chungnam National University Hospital, Daejeon, Republic of Korea; 2 Department of Pediatrics, Incheon Medical Center, Incheon, Republic of Korea; 3 Department of Pediatrics, Hallym University Chuncheon Sacred Heart Hospital, Chuncheon, Republic of Korea; 4 Department of Pediatrics, Inje University Sanggye Paik Hospital, Seoul, Republic of Korea; 5 Department of Pediatrics, Eulji University Hospital, Seoul, Republic of Korea; 6 Department of Pediatrics, Kangwon National University School of Medicine, Chuncheon, Republic of Korea; 7 Department of Pediatrics, Pusan National University Children’s Hospital, Yangsan, Republic of Korea; 8 Department of Pediatrics, Chungnam National University Sejong Hospital, Chungnam National University College of Medicine, Sejong, Republic of Korea; 9 Department of Pediatrics, Dongguk University Ilsan Hospital, Goyang, Republic of Korea; 10 Department of Pediatrics, The Catholic University of Korea Daejeon’s St. Mary’s Hospital, Daejeon, Republic of Korea; 11 Department of Pediatrics, Eulji University Hospital, Daejeon, Republic of Korea; 12 Department of Pediatrics, Wonkwang University Sanbon Hospital, Gunpo, Republic of Korea; 13 Department of Pediatrics, Dankook University College of Medicine, Cheonan, Republic of Korea; 14 Department of Pediatrics, Chonnam National University Hospital, Chonnam National University Medical School, Gwangju, Republic of Korea; 15 Department of Pediatrics, College of Medicine, Chosun University, Chosun University Hospital, Gwangju, Republic of Korea; 16 Department of Pediatrics, Presbyterian Medical Center, Jeonju, Republic of Korea; 17 Department of Pediatrics, Soonchunhyang University Bucheon Hospital, Bucheon, Republic of Korea; 18 Department of Pediatrics, Daegu Catholic University Medical Center, Daegu, Republic of Korea; 19 Department of Pediatrics, School of Medicine, Kyungpook National University, Daegu, Republic of Korea; 20 Department of Pediatrics, Dong-A University College of Medicine, Busan, Republic of Korea; 21 Department of Pediatrics, Wonkwang University School of Medicine, Iksan, Republic of Korea; 22 Department of Pediatrics, Soonchunhyang University Hospital, Gumi, Republic of Korea; 23 Department of Pediatrics, College of Medicine, The Catholic University of Korea, Uijeongbu St. Mary’s Hospital, Uijeongbu, Republic of Korea; 24 Department of Pediatrics, University of Ulsan College of Medicine, Gangneung Asan Hospital, Gangneung, Republic of Korea; 25 Department of Pediatrics, Busan St. Mary’s Hospital, Busan, Republic of Korea; 26 Department of Pediatrics, Sungkyunkwan University School of Medicine, Kangbuk Samsung Hospital, Seoul, Republic of Korea; 27 Department of Pediatrics, Soonchunhyang University Hospital, Seoul, Republic of Korea; 28 Department of Pediatrics, CHA Bundang Medical Center, CHA University School of Medicine, Seongnam, Republic of Korea; 29 Department of Pediatrics, Kogel Hospital, Daejeon, Republic of Korea; 30 Department of Pediatrics, Kwangju Christian Hospital, Kwangju, Republic of Korea; 31 Department of Pediatrics, Pusan National University Hospital, Pusan, Republic of Korea; 32 Department of Pediatrics, Bundang Jesaeng Hospital, Seongnam, Republic of Korea; 33 Divison of Bacterial Diseases, Bureau of Infectious Disease Diagnosis Control, Korea Disease Control and Prevention Agency (KDCA), Sejong, Republic of Korea; 34 Department of Pediatrics, Chungnam National University School of Medicine, Daejeon, Republic of Korea

**Keywords:** Pneumonia, Child, Surveillance, Epidemiology

## Abstract

**Background:**

Respiratory infections among children, particularly community-acquired pneumonia (CAP), is a major disease with a high frequency among outpatient and inpatient visits. The causes of CAP vary depending on individual susceptibility, the epidemiological characteristics of the community, and the season. We performed this study to establish a nationwide surveillance network system and identify the causative agents for CAP and antibiotic resistance in Korean children with CAP.

**Methods:**

The monitoring network was composed of 28 secondary and tertiary medical institutions. Upper and lower respiratory samples were assayed using a culture or polymerase chain reaction (PCR) from August 2018 to May 2020.

**Results:**

A total of 1023 cases were registered in patients with CAP, and PCR of atypical pneumonia pathogens revealed 422 cases of *M. pneumoniae* (41.3%). Respiratory viruses showed a positivity rate of 65.7% by multiplex PCR test, and human rhinovirus was the most common virus, with 312 cases (30.5%). Two hundred sixty four cases (25.8%) were isolated by culture, including 131 cases of *S. aureus* (12.8%), 92 cases of *S. pneumoniae* (9%), and 20 cases of *H. influenzae* (2%). The cultured, isolated bacteria may be colonized pathogen. The proportion of co-detection was 49.2%. The rate of antibiotic resistance showed similar results as previous reports.

**Conclusions:**

This study will identify the pathogens that cause respiratory infections and analyze the current status of antibiotic resistance to provide scientific evidence for management policies of domestic respiratory infections. Additionally, in preparation for new epidemics, including COVID-19, monitoring respiratory infections in children and adolescents has become more important, and research on this topic should be continuously conducted in the future.

**Supplementary Information:**

The online version contains supplementary material available at 10.1186/s12879-022-07263-z.

## Background

Pneumonia is an infection of the lower airways, and community-acquired pneumonia (CAP) specifically refers to clinical signs and symptoms of pneumonia acquired outside a hospital setting [1]. In the United States, CAP is the most common cause of hospitalization among children, with an annual incidence of 15.7–22.5 hospitalizations per 100,000 children [2, 3]. According to statistics from the Health Insurance Review and Assessment Service, nearly half (44.6%) of the 1.59 million pneumonia patients in 2015 were reported to be children under the age of 10, and in 2018, children under the age of 10 often have respiratory system diseases, which are the most common diseases for outpatient and inpatient visits, with pneumonia ranking second among the causes of inpatient visits [4]. These respiratory diseases are mainly caused by bacterial or viral infections, and treatments should be selected depending on the pathogen that causes them. However, clinical symptoms alone cannot distinguish the pathogens, so they should be assessed via laboratory tests. In most CAPs, empirical treatment is provided in that the causes of pneumonia vary widely depending on individual sensitivity, the dynamics of the community, and seasons. It is difficult to obtain samples for microbiological diagnosis. Thus, epidemiological data on clinical patterns and causes of childhood pneumonia will help care, including determining the direction of treatment [5].

The development of the *Haemophilus influenzae* type b (Hib) vaccine and protein binding pneumococcal conjugate vaccine (PCV) has rapidly decreased the incidence of Hib pneumonia and pneumococcal pneumonia [6]. Data on changes in the serologic type of *S. pneumoniae* and antimicrobial susceptibility following the introduction of the pneumococcal vaccine are insufficient. Additionally, the data on pneumonia caused by other bacterial pathogens are insufficient.

Pneumonia by *Mycoplasma pneumoniae* is also a major infectious illness for children in kindergarten and school age, and it continues to be prevalent every 3 to 4 years [7–9]. The incidence of mycoplasma infection increased in 2007, and the incidence rate soared from 2010 to 2011 [7]. *M. pneumoniae* is often accompanied by pleural fluid and shows lobar pneumonia, which is difficult to differentiate from bacterial pneumonia by referring to clinical patterns and chest radiologic findings [8]. Recently, macrolide-resistant *M. pneumoniae* in Korea has been reported, and it is necessary to determine the nationwide status of the disease, the effectiveness of antibiotic treatment, and the degree of resistance.

There have been reports of the distribution of pathogens, epidemic trends, and antibiotic resistance of bacterial pathogens to respiratory infections in adults, but there have been no nationwide reports in children and adolescents in Korea. In particular, CAP in children and adolescents accounts for more than 50% of all cases, the pathogen of pneumonia is different from that of adults, and recent vaccination has resulted in changes in pathogens. For this reason, a nationalized study is needed to analyze the prevalence, epidemic characteristics, and pathogens of pneumonia in children and adolescents. Therefore, the purpose of this study is to establish a monitoring network for CAP among children in connection with community-based cooperative hospitals and the Korea Disease Control and Prevention Agency (KDCA). In addition, we studied the distribution of pathogens, epidemic trends of CAP, and antibiotic usage and resistance of bacterial pathogens.

## Methods

### Patients and study design

A prospective study was conducted in patients under 18 years old who attended a hospital with a diagnosis of CAP. Patients who fulfilled the selection criteria were studied from August 2018 to May 2019 (first research period) and from August 2019 to June 2020 (second research period). The cooperative hospital monitoring network was established for the second and tertiary hospitals in six metropolitan areas (Seoul, Gyeonggi Province, Chungcheong Province, Gangwon Province, Jeolla Province, Gyeongsang Province). From August 2018 to May 2019, 27 hospitals participated, and from August 2019 to June 2020, 28 hospitals participated. The Korean Childhood Community-Acquired Pneumonia Study Group (KoC-CAPS) was established, and this network represented laboratory monitoring networks where pneumonia data could be collected, managed, and analyzed and information could be shared among participating hospitals (Additional file 1: Fig. S1).

### Inclusion criteria

The inclusion criteria were as follows: patients who had a cough or a severe fever as a major symptom, patients diagnosed with pneumonia with a chest X-ray, and patients under 18 years of age who had no history of using antibiotics within 5 days of the visit period.

### Exclusion criteria

The exclusion criteria were as follows: patients with an upper respiratory tract infection that was accompanied by rhinitis symptoms such as a runny nose or stuffy nose, patients who had chronic underlying diseases or immune suppressive disease, and patients with a history of antibiotic use within 5 days.

### Study participants

For each patient, a questionnaire with clinical and epidemiological features was completed. The questionnaire included the following information: patient information (age, birth date, sex, name of the hospital, specimen type, history of hospitalization, underlying disease, vaccination status, siblings), clinical information (fever, cough, sputum, rhinorrhea, vomiting, sore throat, etc., radiologic findings, vital signs, breathing sound, O_2_ saturation), and treatment (prescribed antibiotics, hospitalization period, sequelae) (Additional file 2: Table S1).

### Collection of samples

Samples were obtained from sputum, nasopharyngeal aspiration, nasopharyngeal swab or bronchoalveolar lavage (BAL). Nasopharyngeal aspirates were obtained within 24 h after enrollment. A suction catheter was used to pass through the nose into the lower part of the pharynx. A total of 2 ml nasopharyngeal aspirates were obtained and sent to a laboratory for analysis within 48 h. A nasopharyngeal swab was obtained by inserting a swab into both nostrils parallel to the palate. A throat swab was obtained from the posterior pharyngeal and tonsillar areas. Sputum or BAL specimens were also collected if possible. Viruses [respiratory syncytial virus (RSV) A and B, influenza virus (IFV) A and B, parainfluenza virus (PIV) 1, 2, 3, and 4, adenovirus (ADV), human rhinovirus (HRV), human metapneumovirus (HMPV), coronavirus (CoV) 229E, NL63, OC43, bocavirus (BoV), and human enterovirus (HEV)] were assessed using the multiplex PCR method. Atypical pneumonia pathogens (*M. pneumoniae*, *Chlamydophila pneumoniae*, *Legionella pneumophilia, Bordetella pertussis*) were identified using polymerase chain reaction (PCR) tests and cultures. Cultures for bacterial pathogens (*S. pneumoniae*, *H. influenzae*, *Staphylococcus aureus*, *Klebsiella pneumoniae*, *Pseudomonas aeruginosa*) and antimicrobial susceptibility tests were performed.

### The detection of pathogen

#### A viral pathogen

For multiplex RT-PCR, viral genomic RNA and DNA were extracted from a total volume of 1 µl of the sample by the guanidinium thiocyanate extraction method. The lysis buffer included 500 molecules of the cloned amplified product used as an internal control in each reaction tube and then excluded false-negative results. Three independent multiplex reverse transcription nested RT-PCT assays, able to detect from 1 to 10 copies of viral genomes, were performed. One nested RT-PCR was performed using a specific primer for PIV (1, 2, 3, 4), ADV, HEV, and HMPV, another nested RT-PCR was prepared with specific primers for HRV (A, B, C), CoV (229E, NL63, OC43), and BoV (1, 2, 3, 4), and a third nested RT-PCR was performed using specific primers for RSV (A, B) and IFV (A, B, subtype H1, H3, H1pdm09) by using the Allplex™ Respiratory Panel (Seegene, Seoul, South Korea).

### Atypical pneumonia pathogen

Atypical pneumonia pathogens were detected by PCR. Nucleic acid was extracted from a total volume of 1 ml of the sample and purified. The cyclic temperature settings were 94 ℃ 20 s, 58 ℃ 20 s, and 72 ℃ 20 s amplified by 35 cycles, with the last 72 ℃ 7 min. *M. pneumoniae*, *C. pneumoniae, L. pneumophilia* and *B. pertussis* were confirmed by using the Allplex™ PneumoBacter Assay (Seegene, Seoul, South Korea).

### A bacterial pathogen

Bacterial pathogens were detected by culture, and cultured pathogens were subjected to antimicrobial susceptibility tests. If the final identification of the bacteria was ambiguous or required accurate identification, we confirmed the results by using VITEK 2 (bioMerieux, Hazelwood, USA) to verify the infection, and additional PCR tests were performed for *S. pneumoniae* and *H. influenzae* by using the Allplex™ PneumoBacter Assay (Seegene, Seoul, South Korea).

### The susceptibility test of antibiotics

In this study, the antibiotic susceptibility test of isolated bacteria measured the minimum inhibitory concentration (MIC) using a MicroScan^®^ Microbiology System (Dade Behring, Tokyo, Japan). The criteria for judging antibiotic resistance followed the criteria of the Clinical Laboratory Standard Institute (CLSI).

## Results

### Demographic results

A total of 1023 patients under 18 years old with a diagnosis of CAP were studied. The mean age was 5.0 ± 4.1 years (mean ± standard deviation, range: 1 month–18 years). Most patients were between 1 and 3 years old (310, 30.3%), followed by children between 7 and 11 years old (281, 27.5%), between 4 and 6 years old (210, 20.5%), under 12 months (146, 14.3%), and over 12 years old (75, 7.3%). There was a case in which age entry was omitted. There were 537 males (52.5%) and 486 females (47.5%). Regionally, 360 were reported in Gyeongsang Province, 219 in Chungcheong, 215 in Gyeonggi, 114 in Seoul, 101 in Jeolla, and 14 in Gangwon.

Of the 1023 people, 976 (95.4%) were hospitalized, and 45 (4.4%) were treated in outpatient clinics. There were 2 cases in which hospitalization was unknown. The mean length of hospital stay for the 932 people whose hospitalization period was specified was 6.8 days (range 1–47 days). Specimens were collected from 727 (71.1%) nasopharyngeal aspirate samples, 195 (19.1%) sputum samples, 73 (7.1%) nasopharyngeal swab samples, 12 (0.2%) BAL samples, 8 (0.8%) throat swab samples, and 2 (0.2%) transtracheal aspirate samples. The most frequent symptoms were cough (93.3%), fever (86.1%), sputum (78%), and rhinorrhea (51.1%). The most frequent physical examinations were rale (65%), wheezing (19.5%), decreased aeration (10.5%), and hypoxia (5.2%).

Among the radiological findings of pneumonia, peribronchial infiltration was the most common (44.4%), followed by lobar infiltration (42.8%), interstitial infiltration (11.1%), and pleural effusion (3.8%). There were 212 patients with underlying disease; among them, 79 had an allergic disease, 14 had congenital heart disease, 4 had kidney disease, 4 had endocrine disease, and 2 had cerebrovascular disease. Other accompanying diseases included neurological disorders, Down syndrome, developmental delays, etc. The antibiotic prescription rate was 91.8% (939), and of them, the macrolide prescription rate was the highest (69.6%), followed by cephalosporin (40.8%), and penicillin series (33.2%). All of the penicillin series used were semisynthetic penicillin. There were 84 cases in which antibiotics were not prescribed. The characteristics of the study population are depicted in Table 1.

**Table 1:** Demographic and clinical features of the study population

Mean Age ± SD	5.0 ± 4.1 yrs	Clinical findings	N (%)	Physical examinations	N (%)
Age range (min–max)	1 mo–18 yrs	Cough	992 (93.8)	Rale	688 (65)
Age	N (%)	Fever	911 (86.1)	Wheezing	206 (19.5)
< 12 month	146 (14.3)	Sputum	825 (78)	Decreased aeration	111 (10.5)
1–3 yrs	310 (30.3)	Rhinorrhea	541 (51.1)	Hypoxia < 95%	55 (5.2)
4–6 yrs	210 (20.5)	Poor oral intake	137 (12.9)	Chest retraction	50 (4.7)
7–11 yrs	281 (27.5)	Chill	86 (8.1)	Prescribed antibiotics	N (%)
≧ 12 yrs	75 (7.3)	Sore throat	86 (8.1)	Macrolide	654 (69.6)
Unknown	1 (0.1)	Dyspnea	58 (5.5)	Cephalosporin	383 (40.8)
Total	1,023	GI symptom		Penicillin series	312 (33.2)
Gender	N (%)	Vomiting	70 (6.6)	Tetracyclin	59 (6.3)
Male	537 (52.5)	Diarrhea	37 (3.5)	Quinolone	39 (4.2)
Female	486 (47.5)	Abdominal pain	34 (3.2)	Aminoglycoside	5 (0.5)
Place of treatment	N (%)	Myalgia	36 (3.4)	Vancomycin	3 (0.3)
Hospitalized	976 (95.4)	Chest pain	21 (2)	Lincomycin	3 (0.3)
Outpatient clinic	45 (4.4)	Hoarseness	17 (1.6)	Total	939
Species of sample	N (%)	Rash	11 (1)		
Nasopharyngeal aspirates	727 (71.1)	Hemoptysis	5 (0.5)		
Sputum	195 (19.1)	Chest X-ray finding		N (%)	
Nasopharyngeal swab	73 (7.1)	Peribronchial infiltration		470 (44.4)	
Bronchoalveolar lavage	12 (1.2)	Lobar infiltration		453 (42.8)	
Throat swab	8 (0.8)	Interstitial infiltration		117 (11.1)	
Transtracheal aspirate	2 (0.2)	Pleural effusion		40 (3.8)	

*N* number, *SD* standard deviation, *mo* months, *yrs* years, *GI* gastrointestinal

### Respiratory pathogens

**Fig. 1:**
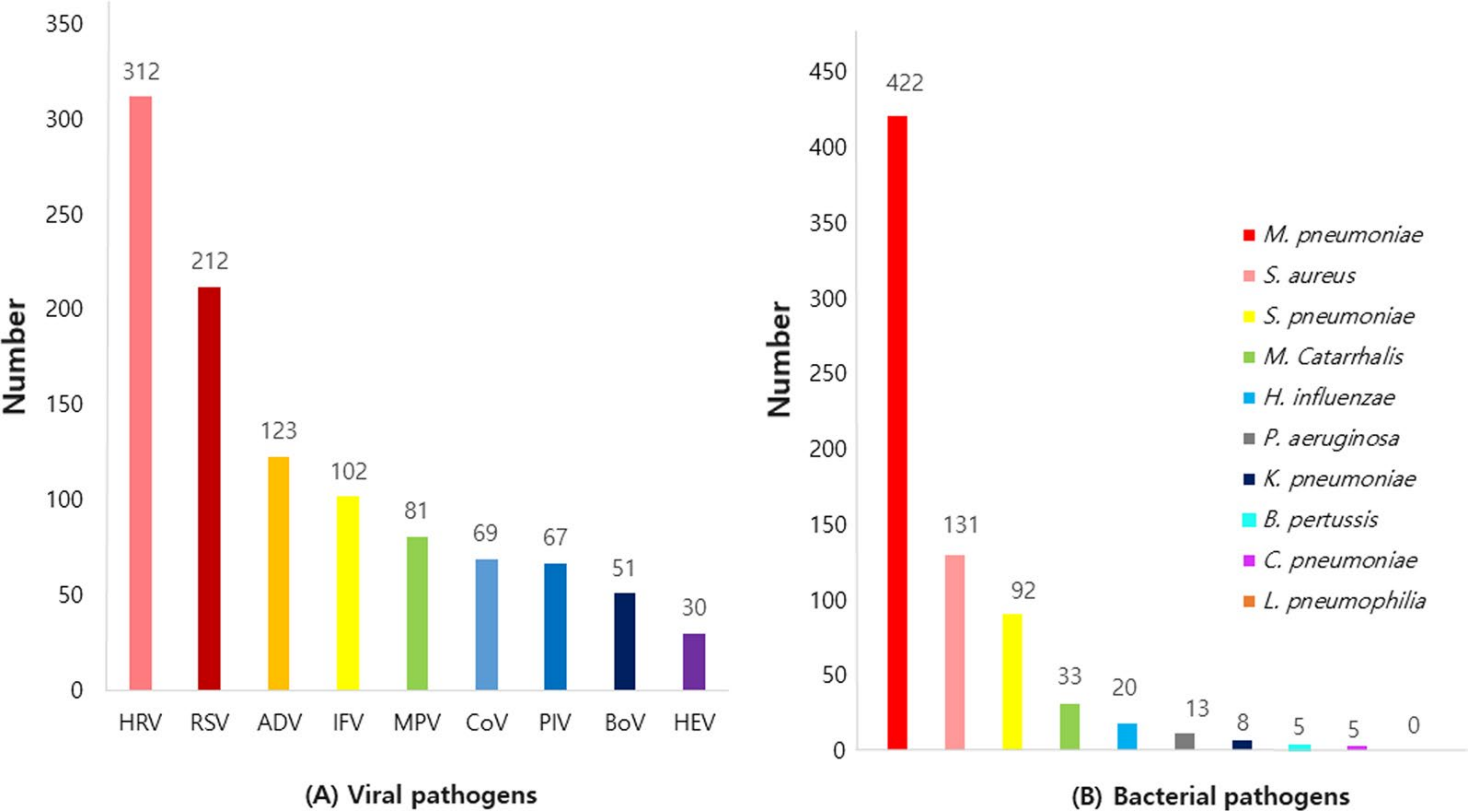
The number of detected respiratory pathogens. **A** Respiratory viral pathogens. **B** Respiratory bacterial pathogens. *HRV  *Human rhinovirus, *RSV* Respiratory syncytial virus, IFV Influenza virus, *PIV* Parainfluenza virus, *ADV * Adenovirus, *HMPV* Human metapneumovirus, *CoV* Coronavirus, *BoV* Bocavirus, *HEV *human enterovirus

A total of 1023 samples of CAP were tested by virus PCR, and the rate of positivity for respiratory viruses was 65.7%. HRV was highest, with 312 (29.8%), followed by RSV (A + B) 212 (20.3%), ADV 123 (11.8%), IFV (A + B) 102 (9.8%), HMPV 81 (7.7%), CoV (OC43 + NL63 + 2229E) 69 (6.6%), PIV (1 + 2 + 3 + 4) 67 (6.4%), BoV 51 (4.6%), and HEV 30 (2.9%) (Fig. 1A). The frequency order of viruses was slightly different when comparing the results of the first and second research periods. The frequency of virus detection changed in the second period; it was ADV, IFV, CoV, HEV, PIV, HMPV, and BoV in the second period, and it was HMPV, ADV, PIV, BoV, IFV, CoV, and HEV in the first period. The number of detection for each virus detected in the sputum are found in Additional file 2: Table S2. Of the 1023 cases, the atypical pneumonia pathogen was identifiable in 432 cases (42.2%) by PCR and the bacterial pathogen was identifiable in 264 cases (25.8%) by culture. Of the atypical bacterial pathogens, there were 422 cases (97.7%) of *M. pneumoniae*, five cases (1.2%) of *C. pneumoniae,* five cases (1.2%) of *B. pertussis,* and *L. pneumophilia* was not found. The bacterial pathogens isolated by culture were as follows. There were 131 cases (12.8%) of *S. aureus*, 92 cases (9%) of *S. pneumoniae*, 33 cases (3.2%) of *Moraxella catarrhalis,* 20 cases (2%) of *H. influenzae*, 13 cases (1.3%) of *P. aeruginosa*, and 8 cases (0.8%) of *K. pneumoniae* (Fig. 1B). In particular, the number of detection for each pathogen found in the sputum are in Additional file 2: Tables S3, S4. The number of bacterial pathogens identified by culture in sputum, nasopharyngeal aspirate and nasal swab, etc., respectively are found in Additional file 2: Table S5.

### Annual and seasonal patterns of respiratory pathogens of CAP

**Fig. 2:**
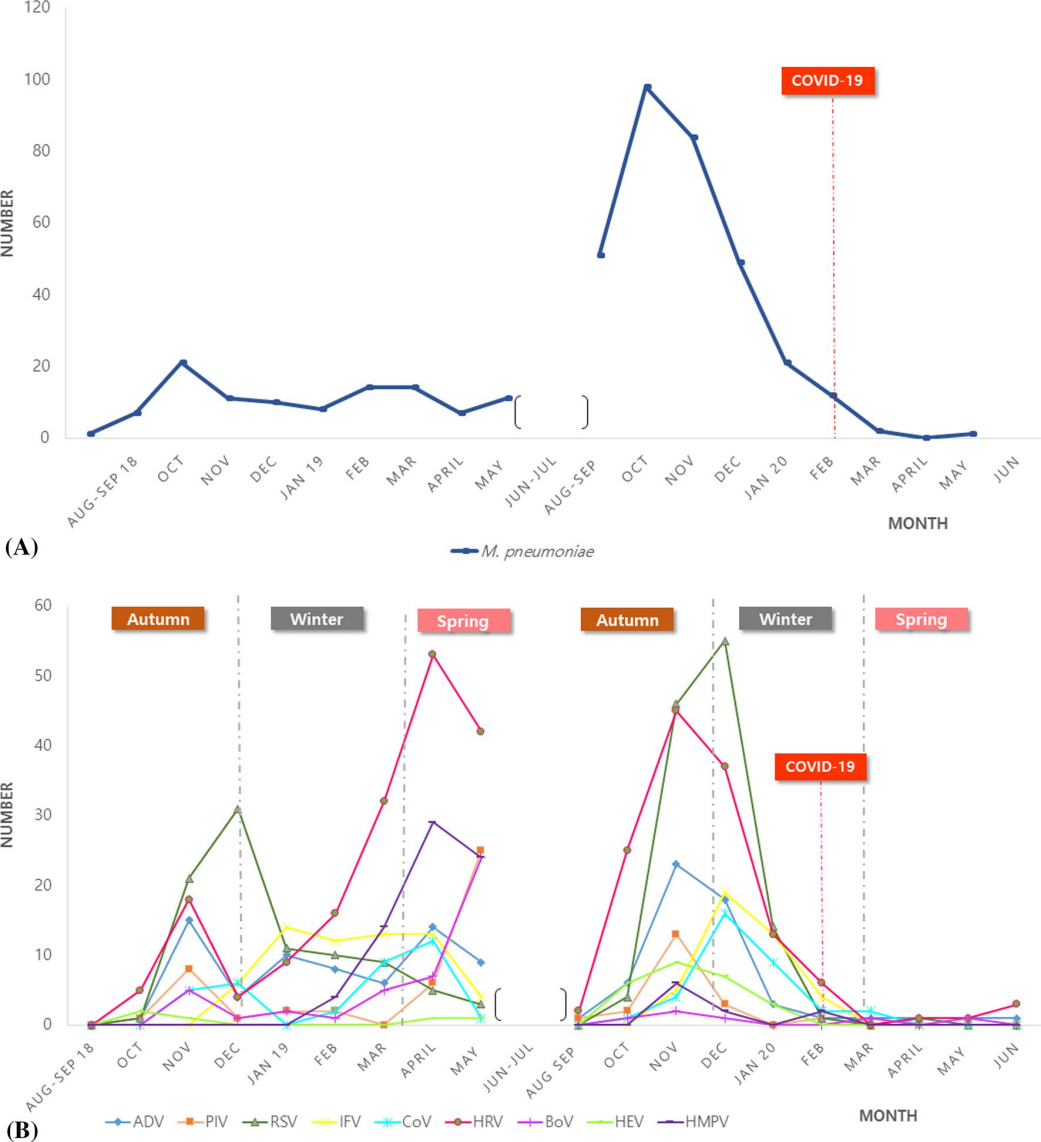
The annual and seasonal patterns of the respiratory pathogen in CAP. **A** *M. pneumoniae*, **B** Respiratory viral pathogens. *CAP* community-acquired pneumonia, *HRV* human rhinovirus, *RSV* Respiratory syncytial virus, *IFV* Influenza virus, *PIV* Parainfluenza virus, *ADV* Adenovirus, *HMPV* human metapneumovirus, *CoV* Coronavirus, *BoV* Bocavirus, *HEV* human enterovirus

*Mycoplasma pneumoniae* was the most common bacterial pathogen that showed a surge infection from August 2019 to December 2019. But *M. pneumoniae* was rarely detected after the COVID-19 outbreak (Fig. 2A). Respiratory viruses showed a seasonal pattern, RSV was detected mostly from November to January, ADV showed a biphasic peak in November and April, HMPV in April and May, HRV a dual peak in the spring and autumn seasons, and BoV in April. Respiratory viruses were rarely detected after the COVID-19 outbreak. Less HRV, ADV, and BoV were detected, but RSV, IFV, PIV, and HMPV were not detected after the COVID-19 outbreak (Fig. 2B).

### Results of respiratory pathogens according to age

Respiratory viruses were the most common pathogen of CAP under the age of 3 years. The rates of viruses and *M. pneumoniae* were similar at the ages of 4–6 years. From 7 years of age, bacteria were predominant for CAP; *M. pneumoniae* was the most common bacterial pathogen. According to the positive rate of *M. pneumoniae* by age, the positive rate was highest at ages 7 to 11 (179 cases), followed by ages 4 to 6 (121 cases), ages 1 to 3 (56 cases), ages 12 years and older (48 cases), and at less than 12 months (14 cases). Five cases of *C. pneumoniae* were detected at 7–11 years of age. Five cases of *B. pertussis* were detected at the ages of 1–3 in three cases and 7–11 in two cases. When comparing the virus detection rate by age, the positive rate was the highest among those under 12 months, followed by those aged 1–3, 4–6, 7–11, and 12 or older (Table 2).

**Table 2:** Results of isolated respiratory pathogen according to age

	< 12 mo	1–3 yrs	4–6 yrs	7–11 yrs	≧ 12 yrs	UK	Total
N of sample	147	309	215	270	73	5	1023
Viruses	127 (86.4)	262 (84.8)	136 (63.3)	111 (41.1)	24 (30.8)	1	661 (64.5)
*M. pneumoniae*	14 (9.5)	56 (18.1)	121 (56.3)	179 (66.3)	48 (65.8)	3	422 (41.3)
*S. aureus*	26 (17.7)	24 (7.8)	24 (11.1)	42 (15.6)	14 (19.2)	1	131 (12.8)
*S. pneumoniae*	24 (16.3)	45 (14.6)	12 (5.6)	7 (2.6)	4 (5.5)		92 (9)
*H. influenzae*	1 (0.7)	11 (3.6)	4 (1.9)	4 (1.5)			20 (2)
*P. aeruginosa*	3 (2)	3 (1)	3 (1.4)	3 (1.1)	1 (1.4)		13 (1.3)
*K. pneumoniae*	3 (2)	1 (0.3)	2 (0.9)	2 (0.7)			8 (0.8)
*C. pneumoniae*				5 (1.9)			5 (0.5)
*B. pertussis*	2 (1.4)	1 (0.3)	1 (0.5)	1 (0.4)			5 (0.5)

*N* number, *mo* months, *yrs* years, *UK* unknown

### Results of co-detection

**Fig. 3:**
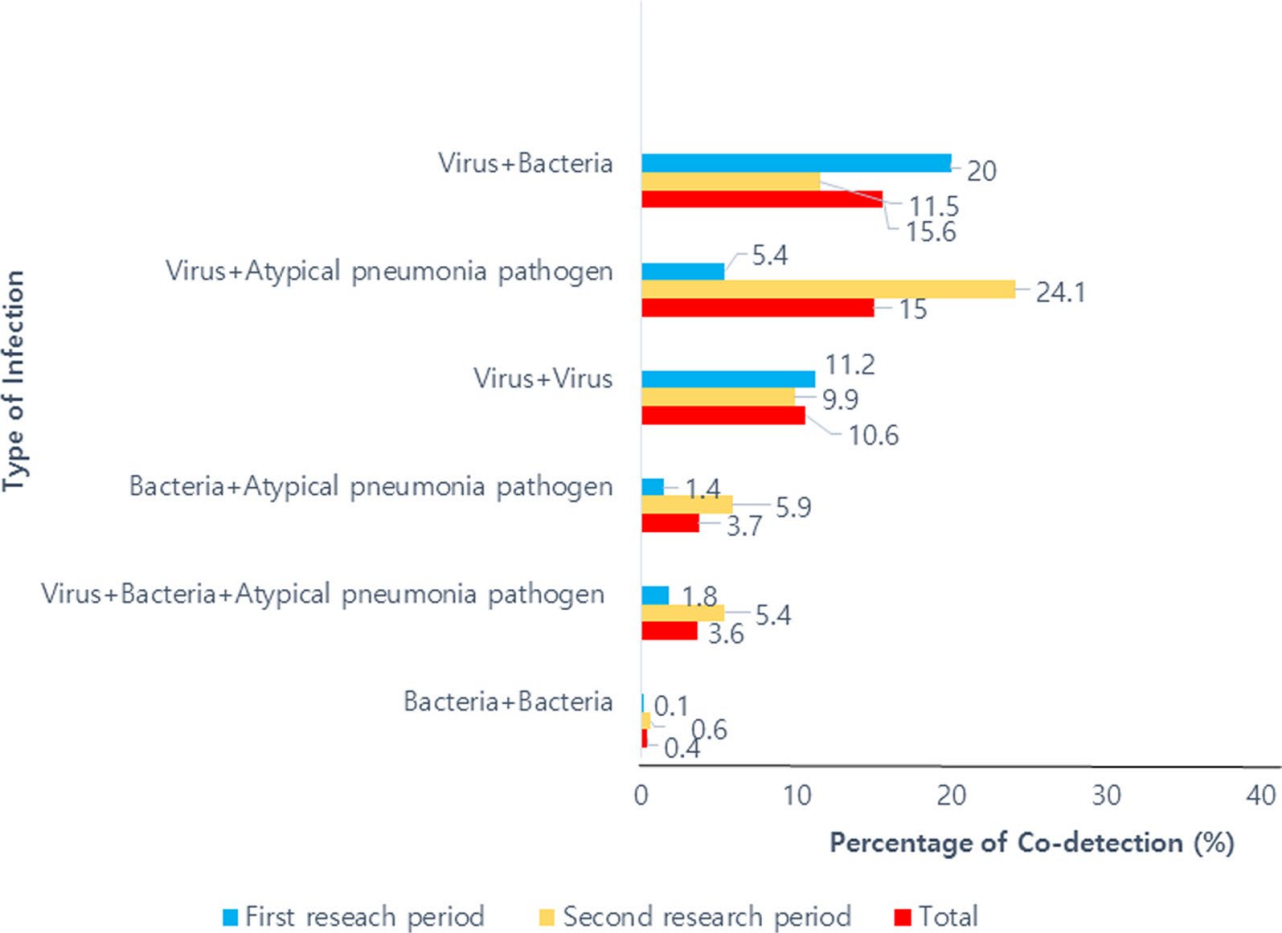
The percentage of co-detection in CAP. *CAP* community-acquired pneumonia

The proportion of co-detection was 49.2%, and the overall rate of co-detection was highest in the virus/bacteria (15.6%), followed by virus/atypical pneumonia pathogens (15%) and virus/virus (10.6%). We analyzed the rate of co-detection in each study period. In the first study period, the co-detection of virus/bacteria (20%) was the highest; however, in the second study period, when mycoplasma pneumonia epidemics occurred, the co-detection of virus/atypical pneumonia pathogens was highest (24.1%) (Fig. 3).

### Resistance to antibiotics

**Fig. 4:**
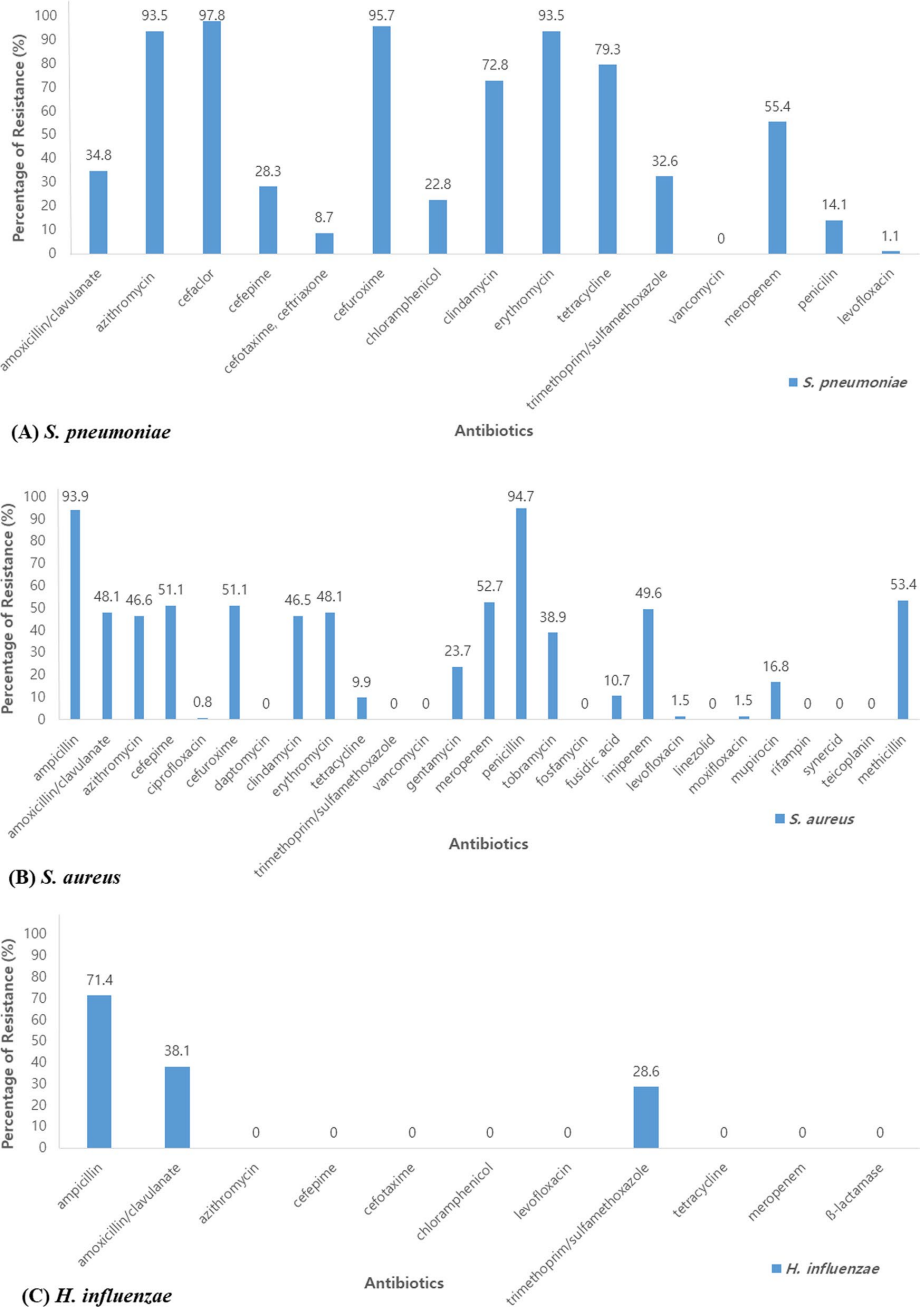

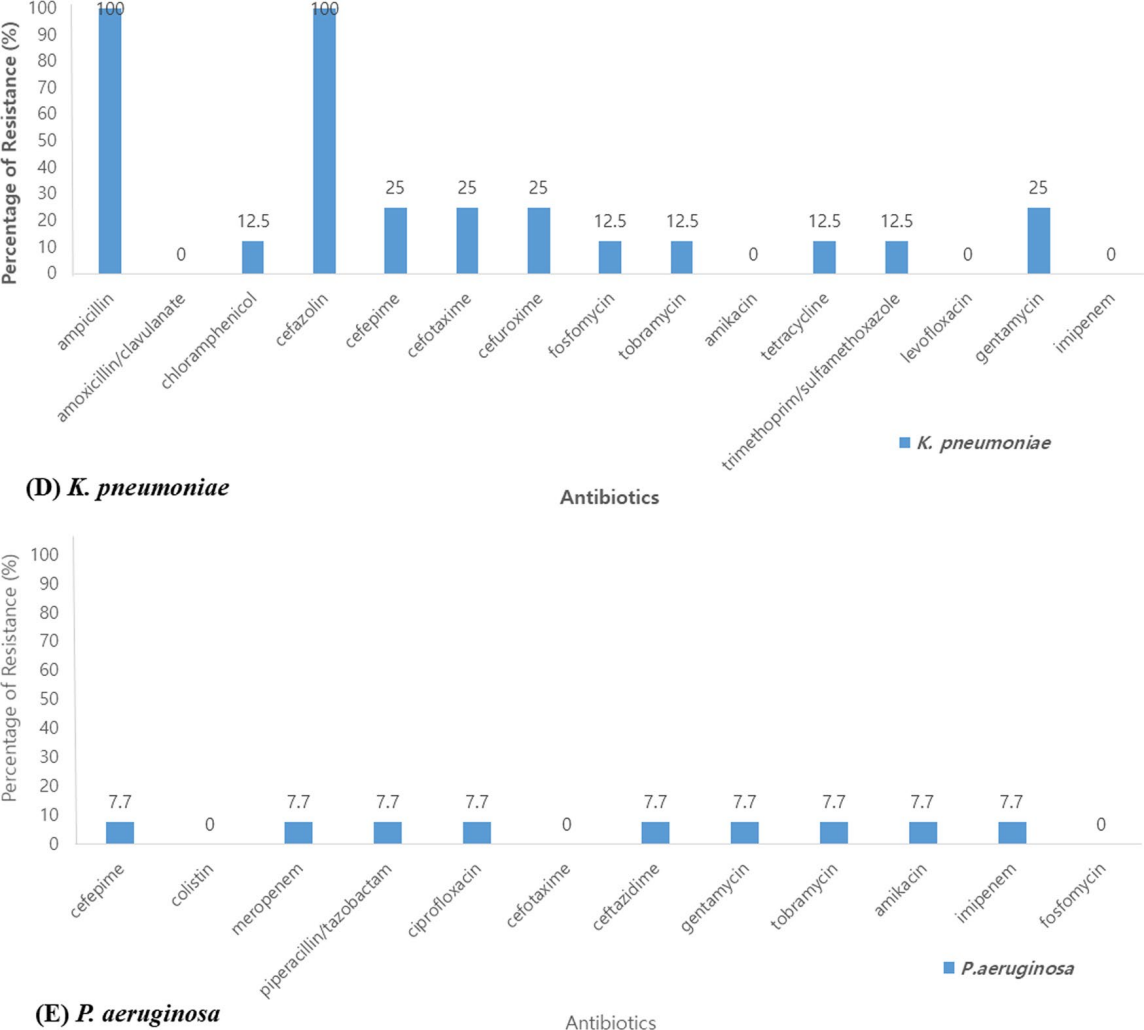
The percentage of resistance in antibiotics. **A** *S. pneumoniae*, **B** *S. aureus*, **C** *H. influenzae*, **D** *K. pneumoniae*, **E** *P. aeruginosa*

We assessed the antibiotic resistance rate of bacteria. Among a total of 92 cases of *S. pneumoniae,* 14.1% were resistant to penicillin, 8.7% to cefotaxime, 93.5% to erythromycin and azithromycin, 79.3% to tetracycline, and 1.1% to levofloxacin. Of 21 cases of *H. influenzae*, 71.4% were resistant to ampicillin, 38.1% to amoxicillin/clavulanate, and no strains were resistant to cefotaxime and tetracycline. Of the 131 cases of *S. aureus*, 94.7% and 53.4% were resistant to penicillin and methicillin, respectively, 48.1% to amoxicillin/clavulanate, 46.6% to azithromycin, 1.5% to levofloxacin, 9.9% to tetracycline, and 46.5% to clindamycin. No strains were resistant to trimethoprim/sulfamethoxazole and rifampin. Of the 8 cases of *K. pneumoniae*, 25% were resistant to cefotaxime, gentamycin and cefepime. No strains were resistant to amoxicillin/clavulanate, levofloxacin and imipenem. Of 13 cases of *P. aeruginosa,* 7.7% were resistant to cefepime, piperacillin/tazobactam, ciprofloxacin, ceftazidime and amikacin. No strains were resistant to colistin (Fig. 4).

## Discussion

Most studies about the causative agents of CAP in children are limited by the difficulty of obtaining adequate specimens. This study is meaningful because it was the first prospective extensive study investigating respiratory pathogens in children with CAP in Korea. In this study, the rate of pathogen detection in children with CAP was 70.5%. However, it could not be confirmed whether the bacterial culture results were the causative pathogen of CAP, and it is highly likely that they were colonized. There are studies about the causative agents of CAP in many countries. In the Taiwanese study, at least one pathogen was identified in 68.3% of children with CAP [10], and in the Chinese study, the causative pathogen was identified in 70.1% of the hospitalized children with CAP at one hospital [11]. In a 3-year US-based study, the causative pathogen was identified in 81% of patients under the age of 18 hospitalized with CAP [2]. In a study conducted in Finland, the causative pathogen was detected in 85% of hospitalized children with pneumonia [12]. The difference in detection rate in each country seems to be due to the difference in sample types and methods. The sample types collected in this study were nasopharyngeal swabs/aspirates and sputum, and the causative pathogens were identified through PCR and cultures. To detect viruses, the same method (PCR test) is used through nasopharyngeal swabs/aspirates in other countries. However, unlike in this study, most tests for bacterial detection were conducted through blood and pleural fluid cultures or blood PCR tests. As a result, it is thought that the detection rate of the pathogen in each study is different.

There are few studies about the causative agent of children with CAP in Korea. In 2009, respiratory viruses were identified in 49.6% of severe lower respiratory tract infections in children [13]. However, unlike in our study, there were no data for bacterial infections. Recently, there was also a study on the seasonal pattern in etiologic viruses and *M. pneumoniae* in children hospitalized with CAP in Korea; it showed that *M. pneumoniae* was the most commonly identified pathogen [14]. It was noticeable that bacterial pathogens as well as viruses were also tested. In general, the common bacterial pathogens of CAP in children are *S. pneumoniae*, *H. influenzae* type b, and *S. aureus.* To differentiate true pathogens from colonization in CAP, cultures from blood/pleural fluid samples or urinary *S. pneumoniae* antigen tests using immunochromatography are better than nasopharyngeal samples [1, 12]. In the current study, we did not use this method; we used bacterial cultures or PCR from nasopharyngeal swabs/aspirates. The detection rate of bacteria from blood cultures in CAP is very low, and pleural cultures are limited to only children with parapneumonic effusion. Urinary *S. pneumoniae* antigen tests can be positive in children if *S. pneumoniae* is colonized in the nasopharynx. *S. pneumoniae* and *H. influenzae* are common flora in the upper respiratory tract, and up to two-thirds of children younger than 5 years are colonized with common bacterial pathogens in the upper respiratory tract. The identification of bacteria from the upper respiratory tract does not always determine the pathogen of CAP; therefore, physicians should consider clinical relevance for ascertaining the bacterial etiologies of pneumonia [15]. It is difficult to identify bacteria that cause bacterial pneumonia, because the introduction of the National Immunization Program (NIP) vaccine has reduced bacterial pneumonia and invasive bacterial infection. Although the results of bacterial culture in the upper respiratory tract sample in children are highly likely to be colonized, there are studies that suggest the association between bacteria colonizing the upper respiratory tract and lower respiratory tract infection in young children [16]. Also, analysis of antibiotic resistance and serotype of bacteria detected in the upper respiratory tract can obtain important information that can be used clinically. Therefore, the present study that reported these results is meaningful.

Over the past 20 years, more than 20 cases of *B. pertussis* have been reported every year in Korea. During 2001–2007, there was an average of 11.3 cases of *B. pertussis* per year [17], and a gradual increase from 2009 to 2012 was reported [18]. In particular, 24.5% of Korean adolescents and adults with chronic cough were positive for *B. pertussis* in 2015 [19]. Due to the lack of data on *B. pertussis* in pediatric patients in Korea, it was important to obtain epidemiological data on *B. pertussis* through the results of the surveillance network study. In this study, *B. pertussis* was isolated from five children, which was less than we expected. All cases were observed between January and April, and two of the patients were younger than 3 months old, while the other three were aged 1, 6 and 9 years old. Due to the NIP by the Korean government, the incidence of *B. pertussis* is low in children, but a gradual increase in adolescents and adults requires the need for a booster injection. Based on these data, it will be necessary to accumulate various epidemiological data on pertussis through the construction of a continuous monitoring network in children and adolescents in Korea.

The common pathogen of CAP in adults was *S. pneumoniae* in a prospective multicenter study in Korea, and in other study, *S. pneumoniae* was most frequent pathogen, atypical pathogens such as *M. pneumoniae,* and *C. pneumoniae* were the second most common pathogens [20, 21]*.* Unlike with adults, the most common cause of CAP in children varies according to age. Several studies have previously reported that respiratory viruses are the leading cause of CAP, which can be detected in more than 50% of the cases [22, 23]. However, these results may vary by study and age group. Similar to our research, in a study of Peru patients under the age of 18 who were hospitalized for pneumonia, *M. pneumoniae* was more frequently detected than respiratory viruses [24]. Additionally, in the Taiwanese study, *S. pneumoniae* was the most common pathogen, and the detection rate of pneumococcus was much higher than that in our study [10]. This may be attributed to relatively lower vaccination rates in Taiwan. Vaccination programs for causative pathogens of pneumonia in Korea are as follows. In 2003, a 7-valent pneumococcal protein-binding vaccine (PCV) was first introduced in Korea, and in June 2010, a 10-valent and a 13-valent PCV were introduced. Currently, Korean government has started pneumococcal vaccination for every infants at 2, 4, 6, and 15 months of age as NIP since 2014, which may rapidly decrease the frequency of *S. pneumoniae* pneumonia. *H. influenzae* type b vaccine is also provided as a NIP since 2013. The Hib vaccine is also given at 2, 4, and 6 months of age as a primary vaccinations and at 12–15 months of age as a booster shot. Pertussis vaccination is also included in a NIP as Diphtheria–tetanus–acellular pertussis vaccine (DTaP) or booster tetanus toxoid, reduced diphtheria toxoid, and acellular pertussis vaccine (Tdap). After three primary vaccinations at 2, 4, and 6 months of age, booster vaccinations are performed at 15–18 months old and 4–6 years old, respectively. In our study, 39 (3.8%) patients were not vaccinated, 86 (8.4%) were unsure whether they were vaccinated. And the rest of subjects (87.8%) were all vaccinated.

The rate of *M. pneumoniae* surged from the fall of 2019, which abruptly ended after the COVID-19 outbreak in 2020. *M. pneumoniae* is a major pathogen of CAP in children and adolescents [9]. Recent studies showed that mycoplasma was responsible for approximately 20–30% of CAP at the age of 3 to 4 years, and during the epidemic *M. pneumoniae* infected even children at 2 years old [5, 9, 25]. *M. pneumoniae* pneumonia epidemics occur every 3 to 5 years [9, 26, 27]. It was reported that epidemics of *M. pneumoniae* pneumonia occurred in 2007, 2011, and 2015 [28, 29]. According to the trend cycle, the results of this study confirmed that the mycoplasma epidemic had been refloating in 2019. Interestingly, the rate of *M. pneumoniae* decreased abruptly after COVID-19. This may be attributed to social distancing, mask-wearing, hand washing, and online schooling.

The seasonal difference in detection rates for viruses is consistent with the existing data. We found that the respiratory viruses were composed of major pathogens for CAP in young children under the age of 3 years. It is known that viruses may cause pneumonia, either directly or by rendering the host more susceptible to bacterial infection [11]. In studies of CAP in children, mixed infections were identified in 34–41% of the children with CAP, and mixed viral-bacterial infection showed the highest rate [11, 30–32]. On the other hand, two US studies reported a lower rate (23–26%) of mixed infection [2, 31]. In this study, the proportion of co-detection was 49.2%, which was higher than in previous studies. The co-detection rate of viruses, bacteria, and atypical bacterial pathogens was 3.6%. It is assumed that the differences in co-detection rate and pattern of co-detection may be related to seasonal, geographical, and racial factors. It may also vary depending on the type of sample or laboratory testing method.

Although viruses are major cause of childhood CAP, a majority of children with pneumonia receive antibiotics. Empiric use of antibiotics remains a cornerstone of treatment in the absence of results in the causative agents of pneumonia. In the U.S. guidelines, amoxicillin is used in previously healthy outpatients who have been properly vaccinated if they suspect mild or moderate bacterial pneumonia. It is recommended to use macrolides if CAP caused by an atypical pneumonia pathogen is suspected [33]. In our study, the empiric antibiotic prescription rate was high, and of them, the macrolide prescription rate was the highest. The high macrolide prescription rate may be attributable to the epidemic of *M. pneumoniae* in 2019.

The rate of antibiotic resistance in this study showed similar results as previous reports. Penicillin sensitivity of pneumococcus in the study of adult pneumonia was 42.9% in 2001 but 100% in 2010 [20, 21]. According to the treatment guidelines of CAP, *S. pneumoniae* has a low resistance rate for penicillin and quinolone and a relatively high resistance rate for some cephalosporin and macrolide [34]. Our results also showed high resistance rates for second-generation cephalosporin and macrolide, while low resistance rates for the third-generation cephalosporin, penicillin, and quinolone, which is similar to what was previously reported. In addition, the results of the antibiotic resistance of *H. influenzae, P. aeruginosa, K. pneumoniae, and S. aureus* were also confirmed to be similar to the 2017 National Antibacterial Resistance Survey Report [35]. These data can be helpful in forming the basis of empiric antibiotic therapy in a child with CAP.

### Study limitations

There were some limitations of this study. First, we could not differentiate true pathogens from normal flora, because we mostly collected samples from the upper respiratory tract. Due to the difficulty in collecting sputum from the lower respiratory tract in young children, this may remain a limitation in children. Second, even though this was a nationwide multicenter study, the number of samples was relatively lower than expected, especially in the outpatient clinic. The number of samples decreased from February 2000. This was the beginning of the COVID-19 epidemic, which led to a decrease in respiratory infections, including in children and adolescents, as well as adults, and the disappearance of respiratory pathogens. In particular, since February 2020, the number of pneumonia patients has decreased sharply, due to restrictions on group activities, as well as high-intensity social distancing, the use of masks, and hand washing. Additionally, the indefinite postponement of openings at kindergarten and schools and the prevalence of homeschooling has also been an important factor in the decrease in infection. Third, laboratory results such as white blood cell count or C-reactive protein were not investigated. In the future, it is necessary to match pathogens with clinical symptoms.

## Conclusions

This study established a surveillance network for monitoring respiratory infections in Korean children. This research provided scientific evidence of policies for managing pneumonia in children and adolescents in Korea by identifying trends in the prevalence of pathogens in children and adolescents with CAP. It will also contribute to the analysis of antibiotic resistance status for bacteria and proper treatment guidelines for children’s respiratory infections in Korea. In addition, this nationwide network system can help to search for a novel pathogen and monitor new respiratory infections such as COVID-19 and provide early national strategies in preparation for a new epidemic.

## Supplementary Information

**Additional file 1: Figure S1.** The Korean Childhood Community-Acquired Pneumonia Study Group; KoC-CAPS.

**Additional file 2: Table S1.** Community acquired pneumonia—Clinical information records. **Table S2.** The PCR results of identified viral pathogen in the sputum. **Table S3.** The PCR results of identified bacterial pathogen in the sputum. **Table S4.** The culture results of identified bacterial pathogen in the sputum. **Table S5.** The number of bacterial pathogens identified by culture in NPA, sputum, nasopharyngeal swab, etc.

## Data Availability

The data were collected through the surveillance system of the Korean Childhood Community Acquired Pneumonia Study Group of the Korean Academy of Pediatric Allergy and Respiratory Disease. Data are available from the corresponding authors upon reasonable request and with permission of the Korean Academy of Pediatric Allergy and Respiratory Disease.

## Abbreviations

CAP: Community-acquired pneumonia
KDCA: Korea Disease Control and Prevention Agency
PCR: Polymerase chain reaction
MIC: Minimum inhibitory concentration
BAL: Bronchoalveolar lavage
HRV: Human rhinovirus
RSV: Respiratory syncytial virus
IFV: Influenza virus
PIV: Parainfluenza virus
ADV: Adenovirus
HMPV: Human metapneumovirus
CoV: Coronavirus
BoV: Bocavirus
HEV: Human enterovirus
Hib: *Hemophilus influenzae* Type b
PCV: Pneumococcal conjugate vaccine
N: Number
SD: Standard deviation
mo: Months
yrs: Years
DTaP: Diphtheria–tetanus–acellular pertussis vaccine
Td: Adult type diphtheria and tetanus toxoid vaccine
Tdap: Booster tetanus toxoid, reduced diphtheria toxoid, and acellular pertussis vaccine
NIP: National immunization program
