# Supplementary figures and images for: Analysis of national surveillance of respiratory pathogens for community-acquired pneumonia in children and adolescents
Source: BMC Infect Dis. 2022 Apr 4;22:330. doi: 10.1186/s12879-022-07263-z (PMC8977558; doi:10.1186/s12879-022-07263-z)

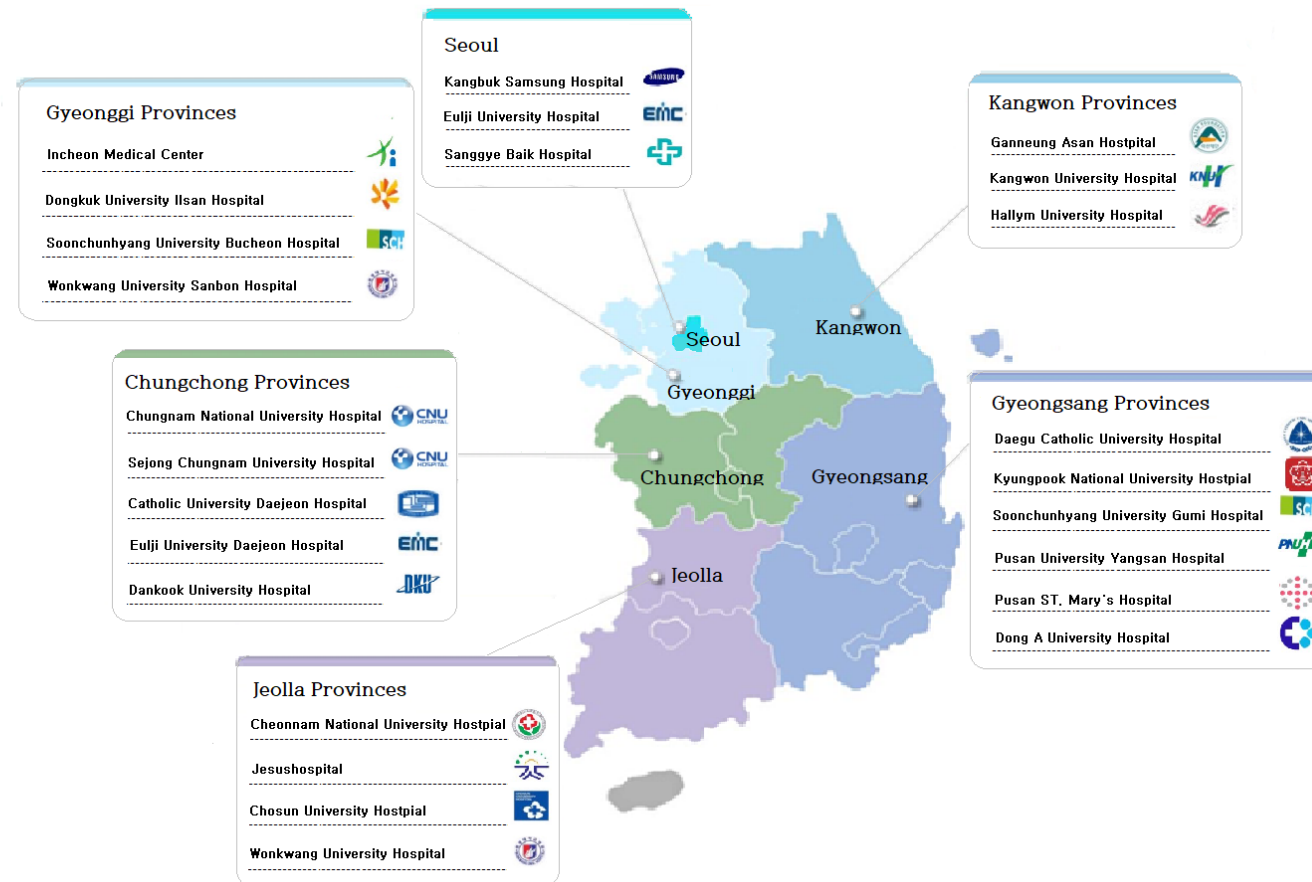

**Figure S1. The Korean Childhood Community Acquired Pneumonia Study Group; KoC-CAPS**

Supplement: Supplementary file 1 — Additional file 1: Figure S1. The Korean Childhood Community-Acquired Pneumonia Study Group; KoC-CAPS. [file 12879_2022_7263_MOESM1_ESM.pdf]
