# Supplementary material for: Analysis of national surveillance of respiratory pathogens for community-acquired pneumonia in children and adolescents
Source: BMC Infect Dis. 2022 Apr 4;22:330. doi: 10.1186/s12879-022-07263-z (PMC8977558; doi:10.1186/s12879-022-07263-z)
Supplement: Supplementary file 2 — Additional file 2: Table S1. Community acquired pneumonia—Clinical information records. Table S2. The PCR results of identified viral pathogen in the sputum. Table S3. The PCR results of identified bacterial pathogen in the sputum. Table S4. The culture results of identified bacterial pathogen in the sputum. Table S5. The number of bacterial pathogens identified by culture in NPA, sputum, nasopharyngeal swab, etc. [file 12879_2022_7263_MOESM2_ESM.pdf]

## Table S1. Community acquired pneumonia -Clinical information records

| | |
|---|---|
| **Name:** | **Physical examination**<br>□ RR(max: ) □ hypoxia(SpO2<95%)<br>□ rale or crackle □ wheezing<br>□ retraction □ decreased aeration |
| **Birth date:** | **Chest X-ray (infiltration)**<br>□ peribronchial □ interstitial<br>□ lobar(RUL, RML, RLL, LUL, LLL)<br>□ pleural effusion(Rt/Lt) |
| **Sex: M/F** | **Antibiotics prescription**: N/Y<br>□ penicillin series<br>□ cephalosporin □ macrolide<br>□ tetracycline □ quinolone □ aminoglycoside<br>□ carbapenem □ glycopeptide<br>□ etc. |
| **Hospital name:** | **Underlying disease**: N/Y<br>□ allergic disease: □ cardiac disease:<br>□ kidney disease: □ endocrine disease:<br>□ etc. |
| **Date of Sampling:** | **Attendence of day care center or school:** N/Y |
| **Species of Sample:**<br>NPA/Sputum/Throat swab/Transtracheal aspirate, BAL, etc | **Sibling**: N/Y |
| **Admission**:N/Y (date of admission: )<br>(date of discharge: ) | **Special consideration:** |
| **Vaccination**:N/Y- DTaP. Td, Tdap, pneumococcus/UK | |
| **Chief Complaint:**<br>Onset: days ago | |
| **Minor Symtoms:**<br>□ fever (duration: days, up to )<br>□ chills □ sore throat<br>□ myalgia □ cough<br>□ sputum □ nasal obstruction<br>□ rhinorrhea □ horseness<br>□ dyspnea □ chest pain<br>□ hemotypsis □ abdominal pain<br>□ vomiting □ diarrhea<br>□ poor oral intake □ rash | |
| **AOM**: N/Y<br>**Pertussis suspicion:** N/Y<br>□ whooping cough □ paroxysmal cough<br>**Mycoplasma pneumonia suspicion** :N/Y | |

Abbreviations: NPA, nasopharyngeal aspirates; BAL, bronchoalveolar lavage; N, no; Y, yes; DTaP, Diphtheria-tetanus-acellular pertussis vaccine; Td, adult type diphtheria and tetanus toxoid vaccine; Tdap, booster tetanus toxoid, reduced diphtheria toxoid, and acellular pertussis vaccine; UK, unknown; AOM, acute otitis media

**Table S2. The PCR results of identified viral pathogen in the sputum**

| Pathogens | N |
|---|---|
| HRV | 44 |
| ADV | 21 |
| RSV | 19 |
| RSV A | 10 |
| RSV B | 9 |
| IFV A | 9 |
| Flu A (H1) | 0 |
| Flu A (pdm09) | 4 |
| Flu A (H3) | 5 |
| IFV B | 0 |
| PIV | 4 |
| PIV 1 | 2 |
| PIV 2 | 0 |
| PIV 3 | 0 |
| PIV 4 | 2 |
| HMPV | 5 |
| CoV | 9 |
| CoV 229E | 3 |
| CoV NL63 | 3 |
| CoV OC43 | 3 |
| HEV | 3 |
| BoV | 1 |
| Total | 124 |

Abbreviations: N, number; HRV, human rhinovirus; ADV, adenovirus; RSV, respiratory syncytial virus; IFV, influenza virus; PIV, parainfluenza virus; HMPV, human metapneumovirus; CoV, coronavirus; HEV, human enterovirus; BoV, bocavirus

**Table S3. The PCR results of identified bacterial pathogen in the sputum**

| Pathogens | N |
|---|---|
| *S. pneumoniae* | 148 |
| *M. pneumoniae* | 136 |
| *H. influenzae* | 123 |
| *C. pneumoniae* | 3 |
| *B. pertussis* | 1 |
| *L. pneumophilia* | 0 |
| Total | 411 |

Abbreviations: N, number

**Table S4. The culture results of identified bacterial pathogen in the sputum**

| Pathogens | N |
|---|---|
| *Streptococcus spp.* | 16 |
| *Staphylococcus aureus* | 8 |
| *Streptococcus parasanguinis* | 5 |
| *Candida albicans* | 5 |
| *Streptococcus oralis* | 5 |
| *Streptococcus mitis* | 4 |
| *Neisseria spp.* | 4 |
| *Streptococcus salivarius* | 3 |
| *Rothia mucilaginosa* | 3 |
| *Serratia marcescens* | 2 |
| *Haemophilus influenzae* | 2 |
| *Pseudomonas aeruginosa* | 2 |
| *Pseudomonas spp.* | 1 |
| *Staphylococcus epidermidis* | 1 |
| *Streptococcus pneumoniae* | 1 |
| *Streptococcus vestibularis* | 1 |
| *Saccharomyces cerevisiae* | 1 |
| *Staphylococcus carprae* | 1 |
| *Candida spp.* | 1 |
| *Staphylococcus lugdunensis* | 1 |
| Total | 72 |

Abbreviations: N, number

**Table S5. The number of bacterial pathogens identified by culture in NPA, sputum, nasopharyngeal swab, etc.**

| | *S. aureus* N | *H. influenzae* N | *S. pneumoniae* N | *K. pneumoniae* N | *P. aeruginosa* N |
|---|---|---|---|---|---|
| NPA | 87 | 15 | 86 | 5 | 10 |
| Sputum | 25 | 3 | 4 | 3 | 3 |
| Nasopharyngeal swab | 17 | 2 | | | |
| Throat swab | 1 | | | | |
| BAL | | | 1 | | |
| Unknown | 1 | | 1 | | |
| Total | 131 | 20 | 92 | 8 | 13 |

Abbreviations: NPA, nasopharyngeal aspirates; BAL, bronchoalveolar lavage; N, number
